# Supplementary material for: Association between grip strength and anthropometric characteristics in the community-dwelling elderly population in Taiwan
Source: PLoS One. 2021 Dec 16;16(12):e0260763. doi: 10.1371/journal.pone.0260763 (PMC8675696; doi:10.1371/journal.pone.0260763)
Supplement: S1 File — Questionnaire using in the survey. (PDF) [file pone.0260763.s001.pdf]

Dear Villagers:
Welcome to participate in the "Comprehensive Community Health Screening" that was organized by the Chiayi County Health Bureau. Under the consideration of personal privacy rights, it is agreed by the local health authority that the data obtained from the screening service and blood sample data will share with the governmental health department (such as the Ministry of Health and Welfare, Taiwan) for evaluation and inquiry or case tracking health management use.

☐ Agree (Signature here)

☐ Disagree

Thanks for your cooperation!
Best regard

Chiayi County Health Bureau
Chang Gung University

(A) Basic demographic characteristics:

1. Birth date: year____, month ____
2. Gender: □(1)Male □(2)Female
3. Ethic: □(1) Aboriginal □(2) Islanders □(3) Hakka □(4) Mainlanders
   □(5)Taiwanese new immigrants : □ ①Vietnamese □ ②Indonesian □ ③Thai
   □ ④Filipino □ ⑤Malaysian □ ⑥Burmese □ ⑦Cambodian
   □ ⑧Chinese □ ⑨Other country _____
4. Marriage status: □(1) Single □(2) Married □(3) Divorce □(4) Widows
5. Education level: □(1) Illiterate □(2) Primary school □(3)Junior high school □(4) Senior high school □(5) College or university
6. Current job:
   (1) □None □ ①Retired ; Previous main occupation before retire____ □ ②House keeper
   □ ③Awaiting job assignment
   (2) □Yes
   □ Agriculture, Fishery and Animal Husbandry □ Soldier □ Government employee
   □ Teacher □ Service industry □ Business □ Hospitality □ Transportation industry
   □ Construction Industry □ Manufacturing □ News Advertising □ Health care industry
   □ Entertainment Industry □ Others _____
   □ Farming fishing
7. Who are your family members who live with (multiple choice)?
   □ Spouse □ Children □ Parents-in-law □ Parents □ Brothers and sisters

(B) General medical history:

Did doctor informed or diagnosed that you have following disease such as [name of the disease]?
Have you regularly been taking medications for chronic disease?

Disease	Have been diagnosed with this disease	Are you currently taking medication
1. Cardiac disease	□0. No □1. Yes	□0. No □1. Yes
2. Arrhythmia	□0. No □1. Yes	□0. No □1. Yes

3. Stroke or cerebral disease	□0. No □1. Yes	□0. No □1. Yes
4. Hypertension	□0. No □1. Yes	□0. No □1. Yes
5. Hyperlipidemia	□0. No □1. Yes	□0. No □1. Yes
6. Diabetes	□0. No □1. Yes	□0. No □1. Yes
7. Gout	□0. No □1. Yes	□0. No □1. Yes
8. Cancer	□0. No □1. Yes<br>If Yes, please fill in the name of the cancer ______________________	□0. No □1. Yes
9. Chronic disease (include under hemodialysis)	□0. No □1. Yes	□0. No □1. Yes
10. Liver disease<br>(1) Liver function impairment<br>(2) Fatty liver<br>(3) Chronic hepatitis B<br>(4) Chronic hepatitis C<br>(5) Liver cirrhosis	(1) □0. No □1. Yes<br>(2) □0. No □1. Yes<br>(3) □0. No □1. Yes<br>(4) □0. No □1. Yes<br>(5) □0. No □1. Yes	(1) □0. No □1. Yes<br>(2) □0. No □1. Yes<br>(3) □0. No □1. Yes<br>(4) □0. No □1. Yes<br>(5) □0. No □1. Yes
11. Chronic pulmonary disease<br>(1) Asthma<br>(2) Chronic bronchitis<br>(3) Emphysema	(1) □0. No □1. Yes<br>(2) □0. No □1. Yes<br>(3) □0. No □1. Yes	(1) □0. No □1. Yes<br>(2) □0. No □1. Yes<br>(3) □0. No □1. Yes
12. Arthritis	□0. No □1. Yes	□0. No □1. Yes
13. Osteoporosis	□0. No □1. Yes	□0. No □1. Yes
14. Other chronic disease<br>(1) _______________<br>(2) _______________	(1) □0. No □1. Yes<br>(2) □0. No □1. Yes	(1) □0. No □1. Yes<br>(2) □0. No □1. Yes

(C) Lifestyle pattern (the following question is asking about your lifestyle in the past 1 year)

1. Do you have the habit of smoking? (Definition: at least smoking once per day for half year)
   □(0) None □(1) Seldon □(3) Often □(4) Quit
2. Does anyone in your family members smoke?
   □(0) None (Skip to question 5) □(1) No member liver with (Skip to question 5) □(2) Yes
3. Will your family members smoke at home?
   □(0) None (Skip to question 5) □(2) Yes
4. Will your family members smoke in front of you? □(0) No □(1) Yes
5. Is your workplace environment a confined space?
   □(0) No (Skip to question 9) □(1) Unemployed (Skip to question 9) □(2) Yes
6. Does anyone smoke in your workplace?
   □(0) None (Skip to question 9) □(1) Yes
7. Will your workplace environment colleagues smoke in front of you?
   □(0) None (Skip to question 9) □(1) Yes

8. Do you take the initiative to avoid smoking when a family member living with you, or a workplace colleague smokes in front of you? □(0) None □(1) Yes
9. Do you currently have a drinking habit? (Definition: at least drinking once per week for half year)
   □(0) None
   □(1) Yes, average ____ times/week
   □(2) Quit
10. Do you currently have a habit of chewing betel nuts?
   □(0) None
   □(1) Yes, begin this habit since Age ___, average ___ Per day
   □(2) Quit
11. How many times do you brush your teeth every day?
   □(0) Almost none □(1) Once every day □(2) At least twice every day
12. Do you brush your teeth before bed/after dinner? □(0) No □(1) Yes
13. Do you use dental floss before bed/after dinner? □(0) No □(1) Yes
14. Number of true teeth ____; Number of dentures _____.
15. How often do you get used to having your teeth washed by a dentist?
   □(0) Almost none □(1) only washed when toothache □(2) only washed when thinking about it
16. Do you have regular health checkups?
   □(0) None □(1) When was the last time? _____ year
   Type : □(1) Regular health check □(2) Elderly health check □(3) Labor Health Check □(4) 3 +1
   □(5) Compound screening □(6) Paid health check □(7) Others_______
17. Do you currently have a habit of exercising regularly? (Definition: at least 3 days/week, 30 mins per day, or total 150 mins / week)
   □(0) None □(1) Yes □(2) Yes, but not now
18. Do you know that the Chiayi County government started to have a health exercise program called "Guarding Your Health in the Home" every day at 7 am and 5 pm on Channel 3 of the Shih Hsin and Dayang? □(0) No □(1) Yes
19. Will you do fitness exercises with this program? □(0) No □(1) Yes
