# Supplementary material for: Association between grip strength and anthropometric characteristics in the community-dwelling elderly population in Taiwan
Source: PLoS One. 2021 Dec 16;16(12):e0260763. doi: 10.1371/journal.pone.0260763 (PMC8675696; doi:10.1371/journal.pone.0260763)
Supplement: Attachment — Submitted filename: Plos-One-Review-Response-20211008.doc [file pone.0260763.s003.doc]

**Oct. 08, 2021**

**Editor-in-Chief
PLOS ONE**

**Dear Editor-in-Chief,**

We would like to thank reviewers for their careful review of our manuscript to provide many valuable comments and suggestions. We have revised and modified our manuscript to fit all reviewers’ requests. Following is the point-by-point changes of our new manuscript. Please forward any comments or suggestions to:

Prof. Nain-Feng Chu

Department of Medicine, Tri-Service General Hospital, Taipei, Taiwan

School of Public Health, National Defense Medical Center, Taipei, Taiwan

PO Box 90048-509, Nei-Hu, Taipei, Taiwan, ROC.

Phone: 886-2-8791-0506, Fax: 886-2-8791-0590

E-mail: [chuepi@mail.ndmctsgh.edu.tw](mailto:chuepi@mail.ndmctsgh.edu.tw); [chuepi369@gmail.com](mailto:chuepi369@gmail.com)

Your Sincerely,

**Nain-Feng Chu, MD, Dr. PH**

Department of Medicine, Tri-Service General Hospital,

School of Public Health, National Defense Medical Center

Taipei, Taiwan, ROC

# **Response to Reviewer 1 Comments**

**Point 1:** Materials and Methods

Interobserver and intraobserver ICC is also needed in all measurements (Grip strength, body waist circumference, etc.).

Additionally, the Grip strength was very important point in this study, the authors should measured a few times. The test-retest reliability (ICC, SEM and minimal difference) is needed.

*Thank you for your valuable comments. We did our best to train our staff to reach the inter-observer variation less than 5% and intra-observer variation around 3-5%.*

**Point 2:** Limitation

Although only one limitation was proposed, it was written as some limitations.

It needs to be revised.

*Thank you for precious suggestion. We had added on some limitations in our update manuscript (Page 10, line 218-220).*

# **Response to Reviewer 2 Comments**

**[Abstract ]**

**Point 1:** Anthropometric is generally recognized as height, weight, body mass index (BMI), body circumference (waist, hips, extremities), sebaceous thickness. However, this study also describes the association between grip strength and medical diseases, so this should be added to the title or modified by changing the title.

*Thank you for your valuable comments. We consider medical diseases as potential confounders and we controlled it in the following analyses. We are focus on the association of anthropometric and GS, we had discussed multimorbidity and GS in other published study (Int. J. Environ. Res. Public Health 2021, 18, 7540. https://doi.org/10.3390/ijerph18147540). But we had revised the title to “Association between grip strength and anthropometric characteristics in the community-dwelling older people” to focus on the population of community elderly in the update manuscript.*

★**Point 2:** Background

The title gives a strong impression that this study focuses on the relationship between grip strength and anthropometric characteristics. However, in the background, the topics of sarcopenia and frailty are discussed. It seems to make it difficult for readers to understand the content. Therefore, if you would like to argue about sarcopenia and frailty, you had better consider as follows: GS and muscle strength, GS and sarcopenia or Frailty, and GS and anthropometric characteristics. These items should be discussed more from the results especially in the discussion section.

*Thank you for your valuable comments. Frailty was usually identified including (1) Weight loss (2) Exhaustion (3) Weakness (decreased grip strength) (4) Slow walking speed (5) Decreased physical activity. (Ref : J Gerontol A Biol Sci Med Sci. 2001;56(3):M146.). Our study was focus on GS and anthropometric characteristics. GS was potentially a key role in diagnosis of sarcopenia and stand for muscle strength in elderly. Besides it was an easier and safe method in a community. To focus on the GS, we had revised this in our update manuscript (as page 2, line 27-31).*

**Point 3:** Design and Method

Please indicate the number of subjects, their age and gender.

*Thank you for your valuable comments. We had revised this in our update manuscript (as page 2, line 34-36).*

**Point 4:** Results

The number of participants and subject details should be described in Design and Method

*Thank you for your valuable comments. We had revised this in our update manuscript (as page 2, line 42).*

**[Introduction]**
★**Point 1:** This is the same as the comments in the abstract.

There was a strong impression that this study focuses on the relationship between grip strength and anthropometric characteristics. However, in the background, the topics of sarcopenia and frailty are discussed. It seems to make it difficult for readers to understand the content. Therefore, if you would like to argue about sarcopenia and frailty, you had better consider as follows: GS and muscle strength, GS and sarcopenia or Frailty, and GS and anthropometric characteristics. These items should be debited from the results especially in the discussion section.

*Thank you for your valuable comments. We had revised these in our update manuscript (as page 4, line 70 and page 9, line 195-203).*

**Point 2:** There should be some kind of conjunction to connect the sentence with the previous sentence as follows: The relationships between sarcopenia, obesity, and central obesity are not well established. This sentence could start with ‘However,….’.

*Thank you for your valuable comments. We had revised this with a drastic revision in our update manuscript (as answer for Point 3).*

**Point 3:** Reference 9 is a research paper on central obesity, sarcopenia, and nutrition. This paper concludes that central obesity and sarcopenia were interrelated with nutritional status in the elderly. However, you state that it is not well established. I would like to know your thoughts or comments on this.

*Thank you for your valuable comments. The results of Ref. 9 (now as Ref. 19) presented a significant association between central obesity, sarcopenia, and nutrition. But some previous study (as Ref. 18) contracted this result. We had adjusted the sentence with a drastic revision and rearrange the sequence of context to avoid possibly confused readers (as page 4, line 66 and line 79-84).*

**Point 4:** The results and discussion are inadequate for the study; similar comments are in the abstract. It needs to be written more coherently. This study aimed to clearly state the epidemiological characteristics of sarcopenia and frailty, but the results also emphasize the relationship between GS and anthropometric parameters, so I think the overall sentence structure needs to be revised in the abstract.

*Thank you for your valuable comments. We had revised these in the update manuscript (as page 5, line 85-91).*

**[Materials and Methods]
Point 1:** Study Populations
Please indicate the number of subjects, their age and gender.
*Thank you for your valuable comments. We had revised these in the update manuscript (as page 5, line 98-100).*

**Poin**t 2: previous three weeks
Simple question, is there any evidence that it is 3 weeks?
*The most common acute illness in community elderly was acute respiratory illness (Ref : https://doi:10.1017/S0950268800050779,* [*https://doi.org/10.1093/infdis/jiz059*](https://doi.org/10.1093/infdis/jiz059)*), and the incubation periods for the upper respiratory tract infection was around 7-10 days, or as long as 21 days (Ref:* [*What are the incubation periods for upper respiratory tract infections (URIs)? (medscape.com)*](https://www.medscape.com/answers/302460-86762/what-are-the-incubation-periods-for-upper-respiratory-tract-infections-uris)*). Thus, we apply 3 weeks as the cut point of inclusion criteria to exclude the possible acute illness related bias for measurement of GS.*

★**Point 3**: Questionnaire
Association between the results of the questionnaire and the results of the anthropometric measurements should be explained in the Discussion. Everything that you have experimented with must be discussed.
*Thank you for your valuable comments. We had revised these in the update manuscript as below. The part of results was re-written on page 8, line 158–167.*

*And the part of discussion about relationships between disease, lifestyles and GS was re-written on page 10, line 208-217.*

**Point 4**: There is insufficient information about the equipment used for the measurement. Only the grip strength tester is listed. Please provide information on all devices.

*Thank you for your valuable comments. We will revise these in the update manuscript (as page 6, line 114-115).*

★**Point 5:** GS measurement
Reference 22, this study examined the relationship between elbow joint angle and grip strength in subjects aged 20-57. Although grip strength is generally measured with the elbow extended. It's unclear about the purpose of measuring it in a sitting position and with the only dominant hand. An explanation or intention for applying this method to the elderly is needed.
*Thank you for your valuable comments. According to the recent Systemic Review article (Mehmet H et. Al. Measurement of hand grip strength in the elderly: A scoping review with recommendations. J Body Move Ther. 2020 Jan; 24(1):235-243. doi: 10.1016/j.jbmt.2019.05.029) which suggests that it may be safer to assess the elderly individual while they are in a seated position.* *Besides, most studies evaluated GS in elderly measured the GS in dominant hand. For safety and universal concern, we applied this method in our study.*

**Point 6**: Statistical methods
I think the grouping cutoff (e.g. normal, weak) should be indicated in the text as well. Also please check the other groupings.
*Thank you for your valuable comments. The definition cut-off value of grip strength was that GS ≥ 30kg in the male and ≥ 20 kg in the female. Weak GS was defined as GS < 30 kg in the male and GS < 20 kg in the female (from the definition of European Working Group on Sarcopenia in Older People, EWGSOP). We had revised these and provide this information in the section of method in the update manuscript (as page 6, line 125-129).*

[Results]
★**Point 1:** I don't think it is necessary here to restate subject details such as age; in Results, you should simply state the results of your analysis.
*Thank you for your valuable comments. We will revise these in the update manuscript (as page 7, line 149).*

**Point 2**: Need to include the results of medical disease risk from blood samples.
*Thank you for your valuable comments. We had revised these in the update manuscript. However, we use the content of questionnaire to evaluate the history of medical diseases.*

**Point 3**: In the sentence as follows; “compared with other anthropometric measures, only WHR showed a negative correlation, but the difference was without or borderline statistical significance in both sexes (r = -0.013, p = 0.613 in males and r = -0.047, p = 0.030 in females)”. For females, the significance is lower, but it is 0.03. I think you need to modify the explanation method.
*Thank you for your valuable comments. We will revise these in the updated manuscript (as page 8, line 171-173)*

**Point 4:** The reason for Model 1 and Model 2 classification is unclear. I do not understand on what basis the models were classified. I think it would be better to indicate whether the classification was based on controllable factors such as congenital or acquired factors.
*Thank you for your valuable comments. We adjust for the demographic and baseline disease history in “Model 1” as general adjusting model. In Model 2, we more focus on the variation of each anthropometric variable to do our best to reduce the potential confounding effects.*

**[Discussion]**
★**Point 1:** Insufficient consideration of measurement results. As commented in the questionnaire section, everything that has been experimented with should be discussed.
*Thank you for your valuable comments. We also discuss about the relationships of GS between medical diseases and habits. We will revise these in the updated manuscript (as page 10, 208-217)*

**Point 2:** In the sentence as follows; “In the current ageing society, sarcopenia and physical disability among the elderly population is becoming increasingly important”. What is important? How is it important? Please explain.
*Thank you for your valuable comments. We will revise these in the updated manuscript (as page 9, line 194-197)*

**Point 3:** In the sentence as follows; “although no obvious and clear mechanism has been discovered”. It is unclear from this what has not been clarified.
*Thank you for your valuable comments. We had revised these sentences with a drastic revision to avoid possibly confused reader (as page 9, line 199-202).*

★**Point 4:** In the sentence, as follows; “This study was the first large community-observed prospective study to investigate the relationship between anthropometric characteristics and GS among individuals older than 65 years in Taiwan”. A poor match between title and research content. This is the same as the comments in the abstract.
*Thank you for your valuable comments. We had revised the sentences of main text with a drastic revision according to your suggestions from the section of abstract to discussion and focus on the relationships between GS and common anthropometric profile (as page 5, line 87-91; page 10, line 230-232; and page 11, line 235 -241).*

**Point 5:** In the sentence as follows; “This result can be explained by the fact that taller individuals also have longer bones, which gives them greater GS”, Reference 32 is a twin study. It shows that GS changes with growth, but I don't think it says that " long bones have stronger GS". It is good to list the literature, but I think this sentence should be deleted or changed to another way of explanation.
*Thank you for your valuable comments. We had revised these and deleted the sentence as your suggestion in the update manuscript (as page 11, line 228).*

★**Point 6:** In the sentence, as follows; “This study applied simple personal anthropometric profile〜”. What you have shown in this paragraph is a unique result. If you want to emphasize the novelty here, you should include more discussion about this.
*Thank you for your valuable comments. We had revised these in the update manuscript (as page 10, line 231-233).*

**[Conclusion]
Point 1:** In the sentence as follows; “To conclude, our study showed that GS in the elderly Taiwanese population was similar to reports on GS in other Asian groups and was weaker than the GS of Western populations”. The term "Taiwanese elderly" is very broad, so I think it would be better to change the wording. Also, which report is being compared to be lower than the Western population?
*Thank you for your valuable comments. We will revise our title and change these in the update manuscript.*

*The GS in Asian was weaker than Western population with ethnic difference was proven in the research as below:*

*1. Anjum SN, Choudary P, Dimri R, Ankarath S. Comparative evaluation of grip and pinch strength in an Asian and European population. Hand Therapy. 2012;17(1):11-14. doi:10.1258/ht.2011.011023*

*2. Ntuk UE, Celis-Morales CA, Mackay DF, Sattar N, Pell JP, Gill JMR. Association between grip strength and diabetes prevalence in black, South-Asian, and white European ethnic groups: a cross-sectional analysis of 418 656 participants in the UK Biobank study. Diabet Med. 2017 Aug;34(8):1120-1128. doi: 10.1111/dme.13323. Epub 2017 Feb 23. PMID: 28144980.*

*We had added these studies as references for the sentence “Ethnic differences were found in GS, and GS was higher in a Western population when compared to an Asian population” (as page 10, line 208).*

★**Point 2:** About the sentence as follows; “muscle weakness among elderly population in Taiwan.” It said, it predicts muscle weakness, but in your discussion, you say, "GS is considered an essential parameter in clinical settings to assess multiple risk factors such as cardiovascular and physical disorders." 1 be better to describe not only the prediction of muscle weakness but also the relationship with other parameters.
*Thank you for your valuable comments. We had revised and adjusted these in the update manuscript (as page 11, line 250-252).*

**[Tables 1, 2, 3, 4]
Point 1:** I think it would be better to show the cutoff values for Normal and Weak, and I have made the same comment in Statistical methods.
*Thank you for your valuable comments. We will revise these in the update manuscript (as Table 1).*

**Point 2:** In Tables 2 and 4, there are no asterisks in the tables for p-values. 1A and 1B have asterisks, and there is a supplementary note below the tables saying **p<0.01, *p<0.05. Therefore, I think it should be standardized for all tables.

*Thank you for your valuable comments. We will revise these in the update manuscript (as Tables 2, 3 and 4).*
