# Supplementary material for: Association between grip strength and anthropometric characteristics in the community-dwelling elderly population in Taiwan
Source: PLoS One. 2021 Dec 16;16(12):e0260763. doi: 10.1371/journal.pone.0260763 (PMC8675696; doi:10.1371/journal.pone.0260763)
Supplement: Attachment — Submitted filename: Plos-One-Review-Response-20211028.doc [file pone.0260763.s004.doc]

**Oct. 28, 2021**

**Editor-in-Chief
PLOS ONE**

**Dear Editor-in-Chief,**

We would like to thank reviewers for their careful review of our manuscript to provide many valuable comments and suggestions. We have revised and modified our manuscript to fit all reviewers’ requests. Following is the point-by-point changes of our new manuscript. Please forward any comments or suggestions to:

Prof. Nain-Feng Chu

Department of Medicine, Tri-Service General Hospital, Taipei, Taiwan

School of Public Health, National Defense Medical Center, Taipei, Taiwan

P.O. Box 90048-509, Nei-Hu, Taipei, Taiwan, ROC

Phone: 886-2-8791-0506, Fax: 886-2-8791-0590

E-mail: [chuepi@mail.ndmctsgh.edu.tw](mailto:chuepi@mail.ndmctsgh.edu.tw); [chuepi369@gmail.com](mailto:chuepi369@gmail.com)

Your Sincerely,

**Nain-Feng Chu, MD, Dr. PH**

Department of Medicine, Tri-Service General Hospital,

School of Public Health, National Defense Medical Center

Taipei, Taiwan, ROC

# **Reviewer #1 Comment**

Interobserver and intraobserver ICC is also needed in all measurements (Grip strength, body waist circumference, etc.). Additionally, the Grip strength was very important point in this study, the authors should measured a few times. The test-retest reliability (ICC, SEM and minimal difference) is needed.

It should be described in the Material and Methods and shown to the reader, not just the reviewer. It is necessary for the reader to judge whether the measurement was performed under appropriate condition.

*Response: Thank you for your valuable comments. We had trained our staff before conduct survey. All measurements of anthropometric variables and grip strength reach the inter-observer variation less than 5% and intra-observer variation less than 3% to ensure the quality.*

*For grip strength, the test-retest reliability of SEM is around 0.2-0.3 and minimal difference is 0.1 kg.*

*We had revised and added this in our update manuscript.*

# **Reviewer #2 Comments**

In my last review, I had asked for a correction regarding the p-value. In that case, for example, in Table 2, only one part was marked with an asterisk. Therefore, it was suggested that it would be better to unify them. In this case, using Table 2 as example, all women are marked with a symbol, while men are not. The intention of the last review was that unification meant that if you wanted to add a symbol, you would put it on everything, and if not, you would remove it all. This is a decision based on your thoughts. Thanks you for your consideration.

*Response: Thank you for your valuable comments. We had added the asterisk symbols in Table 2 (women) and Table 4 (Model I) which would be more standardized and unification for all Tables.*
